# IL-2-driven CD8^+^ T cell phenotypes: implications for immunotherapy

**DOI:** 10.1016/j.it.2023.09.003

**Published:** 2023-10-10

**Authors:** Veronika Niederlova, Oksana Tsyklauri, Marek Kovar, Ondrej Stepanek

**Affiliations:** 1 Laboratory of Adaptive Immunity, Institute of Molecular Genetics of the Czech Academy of Sciences, Prague, Czech Republic; 2 Laboratory of Tumor Immunology, Institute of Microbiology of the Czech Academy of Sciences, Prague, Czech Republic

## Abstract

The therapeutic potential of interleukin (IL)-2 in cancer treatment has been known for decades, yet its widespread adoption in clinical practice remains limited. Recently, chimeric proteins of an anti-PD-1 antibody and suboptimal IL-2 variants were shown to stimulate potent antitumor and antiviral immunity by inducing unique effector CD8^+^ T cells in mice. A similar subset of cytotoxic T cells is induced by depletion of regulatory T cells (Tregs), suggesting IL-2 sequestration as a major mechanism through which regulatory T cells suppress activated CD8^+^ T cells. Here, we present our view of how IL-2-based biologicals can boost the antitumor response at a cellular level, and propose that the role of Tregs following such treatments may have been previously overestimated.

## IL-2 in T cell biology and immunotherapy

IL-2 is a key signaling glycoprotein produced by antigen-activated T cells, which promotes T cell survival, proliferation, and differentiation. T cell antigen receptor (TCR) as well as IL-2 receptor (IL-2R; see Glossary) signaling induces the expression of IL-2Rα (CD25), which is a part of the high-affinity trimeric IL-2 receptor [1]. This constitutes a potent amplification of the IL-2-mediated boost, which is more important for cytotoxic CD8^+^ T cells than for helper CD4^+^ T cells [2–4]. Due to the unique biological properties of IL-2, various IL-2-based immunotherapies have been considered for treating cancer and autoimmune diseases (Box 1) [5,6].

Recently, several studies combined IL-2 agonists with immune checkpoint therapies to treat cancer and chronic infections in preclinical studies [7–11]. Here, we focus on these emerging findings to present our view whereby we argue that a major mechanism of IL-2-mediated immunotherapy is the alleviation of Treg-mediated suppression and the induction of superior cytotoxic CD8^+^ T cells.

## Targeting intermediate-affinity IL-2 on PD-1+ T cells has a potent antitumor effect

**Figure 1:**
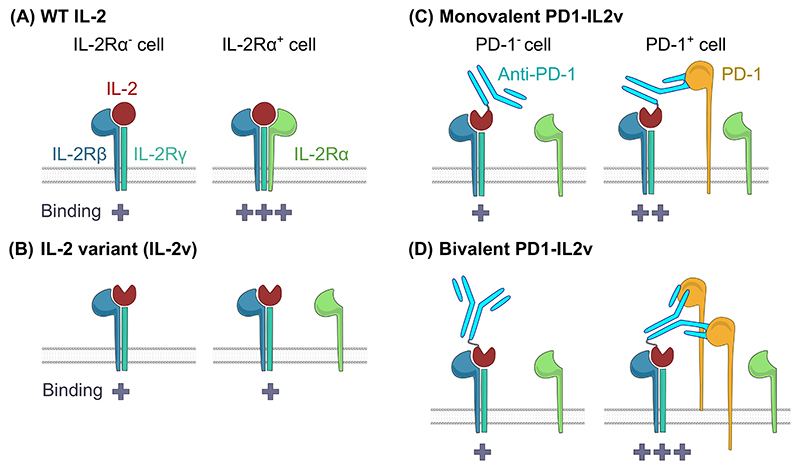
Interleukin (IL)-2 and IL-2 modifications used in immunotherapy approaches. (A) Wild-type (WT) IL-2 can bind to dimeric intermediate-affinity IL-2 receptor (IL-2Rβγ) or trimeric high-affinity IL-2 receptor (IL-2Rαβγ) [1]. (B) Multiple IL-2 variants (IL-2v) were designed with a mutated IL-2Rα-binding site. These variants can bind only to IL-2Rβγ receptors, regardless of the availability of IL-2Rα [48–51,59]. (C,D) The apparent affinity of binding of IL-2 variants (IL2v) to IL-2Rβγ is increased in *cis* by fusion to (C) monovalent [7] or (D) bivalent [8,11] anti-PD-1 antibodies, regardless of the availability of IL-2Rα. These fusion proteins can impede PD-1 inhibitory signaling to some extent [8]. When PD-1 is not expressed, PD1-IL2v can still bind to IL-2Rβγ, but with lower affinity. ‘+’ indicates the binding affinity of IL-2v to IL-2R.

Clinical usage of IL-2 is limited by not only severe adverse effects caused by off-target stimulation of endothelial cells in lungs, brain, and liver [12–14], but also its short *in vivo* half-life [15]. Moreover, until recently, the paradigm in the field proposed that IL-2Rα-binding IL-2-based molecules, such as wild-type (WT) IL-2, predominantly stimulate Tregs and, thus, are largely tolerogenic, whereas IL-2 variants with disabled/limited IL-2Rα interaction preferentially stimulate cytotoxic CD8^+^ T cells and natural killer (NK) cells (Figure 1A,B). Accordingly, multiple engineered variants of IL-2-based therapeutics, such as IL-2 immunocomplexes (IL2ICx), IL-2 immunocytokines, or IL-2 muteins, were developed to avoid the adverse effects of IL-2 and to target only one arm of the immune system (i.e., cytotoxic CD8^+^ T and NK cells for anti-cancer treatment or Tregs in the treatment of autoimmune diseases) (reviewed in [16]).

Immune checkpoint inhibitors represent a major advance in cancer immunotherapy [17]. However, because ICIs are efficient only in a subset of patients, combination therapies are being tested, including the potential synergy of PD-1 blockade and IL-2R agonists [18–20]. Several recent studies showed the robust antitumor effects of IL-2 variant (IL-2v), an engineered variant of IL-2 not interacting with IL-2Rα, which is fused to monovalent or bivalent anti-PD-1 antibody (PD1-IL2v) in various preclinical tumor models (orthotopic pancreatic adenocarcinoma Panc02-H7-Fluc [8] or PK5L1940 [10] in C57/BL6 mice; spontaneous pancreatic tumors in RIP1-Tag5 transgenic C57BL/6 mice [9,10]; subcutaneous A20 lymphoma and Renca adenocarcinoma in BALB/C mice; and MC38 carcinoma in C57BL/6 mice [7], as well as orthotopic GL261 glioma in C57BL/6 mice [9]) (Figure 1C,D).

Blockade of PD-L1, together with WT IL-2 or PD1-IL2v, but not with nontargeted IL-2v, reduced the titers of lymphocytic choriomeningitis virus (LCMV) clone 13 in the spleen and lungs of infected mice during the chronic phase of the disease, relative to no treatment or PD-L1 blockade only [8,11]. Blockade of IL-2Rα disrupted the effect of WT IL-2 in this model [11]. These experiments showed that the binding of IL-2v to the dimeric receptor IL-2Rβγ was sufficient to induce a therapeutic effect only if it was anchored to target T cells in *cis*, and argued for the importance of the trimeric engagement of IL-2R. It is not clear whether IL-2R anchoring needs to be via PD-1 or whether other T cell surface proteins would work in a similar manner.

Based on the above-mentioned studies [7–11], which used various mouse models of cancer and chronic infection, we propose that PD1-IL2v has multiple potential molecular mechanisms of action: (i) targeting of IL-2v to PD-1^+^ tumor- or virus-specific T cells; (ii) strong IL-2Rα-independent binding to IL-2R via its anti-PD-1 antibody-mediated targeting to the cell surface in *cis;* (iii) prolonged interaction with IL-2R via anchoring to PD-1 and slow internalization of the whole complex leading to the removal of PD-1 from the cell surface, as shown in human CD4^+^ T cells [8]; and (iv) PD-1 blockade. The inhibition of PD-1 signaling via PD1-IL2v appears to be only partial, because the addition of a blocking antibody to PD-L1 on top of PD1-IL2v further increases the antitumor effect in pancreatic adenocarcinoma in mice [7,9], as well as the antiviral effect in chronic LCMV infection in mice [8].

Collectively, these studies attribute a candidate therapeutic potential for IL-2-based drugs largely to the stimulatory effects of this cytokine on cytotoxic CD8^+^ T cells, particularly when combined with checkpoint blockade [7–11], although further preclinical and clinical testing is warranted.

## IL-2-based therapy can induce ‘better effectors’: a unique subset of cytotoxic T cells

**Figure 2:**
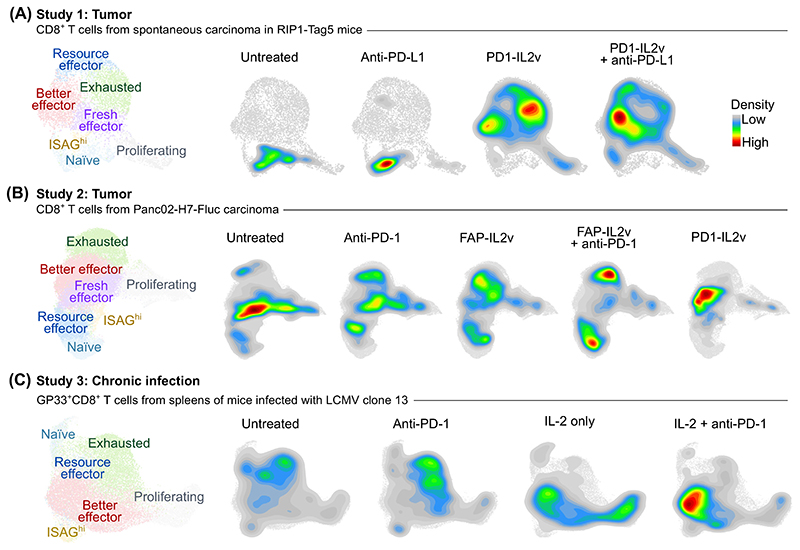
Generation of ‘better effector’ CD8^+^ T cells upon PD-1–interleukin (IL)-2v treatment in mice. (A,B) Uniform manifold approximation and projection (UMAP) plots based on single cell RNA sequencing (scRNAseq), constructed for illustration purposes only, showing (A) CD8^+^ T cells isolated from pancreatic carcinoma in RIP1-Tag5 mice [9], (B) CD8^+^ T cells isolated from subcutaneous Panc02-H7-Fluc pancreatic carcinoma in C57BL/6 mice [8], (C) antigen-specific GP33^+^CD8^+^ T cells isolated from spleens of C57BL/6 mice chronically infected with lymphocytic choriomeningitis virus (LCMV) clone 13 [11]. UMAP plots on the left show the localization of different subpopulations in the dimensional reduction space. UMAP plots on the right show the density of cell populations upon different treatments. ScRNAseq data were obtained from the following studies: Study 1 [9] (GSE197854), Study 2 [8] (E-MTAB-11773), and Study 3 [11] (GSE206739).

IL-2 enhances differentiation and cytotoxic effector cell formation in the CD8^+^ T cell compartment [21]. However, single cell RNA sequencing (scRNAseq) of tumor-infiltrating lymphocytes from murine subcutaneous Panc02-H7-Fluc tumors revealed that PD1-IL2v treatment also increases the frequency of a population of ‘better effector’ T cells (CD8^+^ GZMB^+^ TIM-3^−^ PD-1^+^ TCF7^low/−^) [8]. Similarly, PD1-IL2v treatment increased the frequency of better effector T cells in another tumor model using RIP1-Tag5 transgenic mice, which spontaneously develop solid tumors resistant to immune checkpoint blockade [9]. Taken together, better effector T cells canexpand upon treatment with PD1-IL2v [8,9] (Figure 2A,B, data shown for illustrative purposes only). Accordingly, the combination of anti-PD-1 antibody and IL-2 treatment in chronic LCMV infection induced the formation of better effectors in splenic CD8^+^ T cells [11] (Figure 2C, data shown for illustrative purposes only). These findings suggest that PD1-IL2v induces a unique gene expression program in CD8^+^ T cells, which leads to the formation of a subset with superior effector functionality in certain cancers and chronic infection.

**Figure 3:**
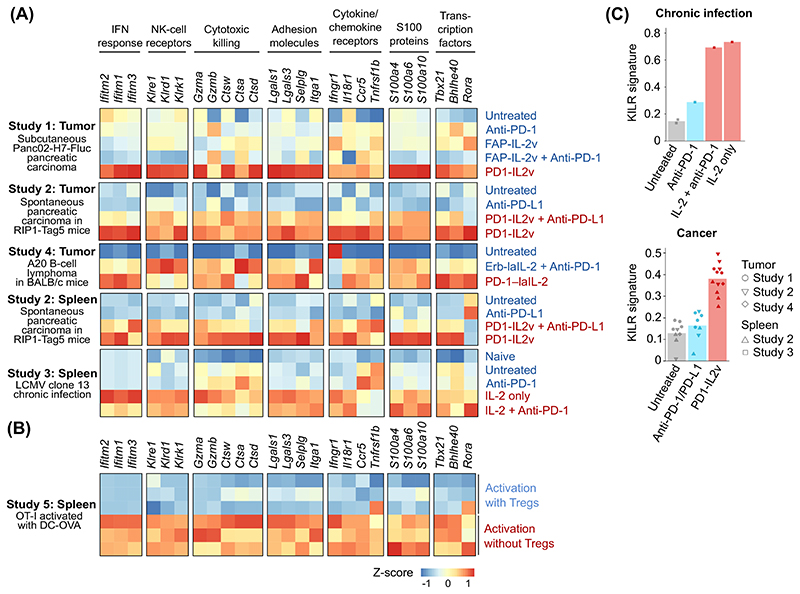
Gene expression signatures of CD8^+^ T cells in response to interleukin (IL)-2 based therapies. (A,B) Heatmaps showing the relative gene expression of selected genes in splenic or intratumoral mouse CD8^+^ T cells, constructed for illustration purposes only: (A) upon treatment with IL-2-based compounds [wild-type (WT) IL-2 [11], PD-1-laIL-2 (intermediate-affinity IL-2 conjugated to anti-PD-1 monoclonal antibodies; mAbs; monovalent) [7], Erb-laIL-2 (intermediate-affinity IL-2 conjugated to anti-EGFR mAb that serves as a control conjugate to PD-1-laIL-2 [7]), FAP-IL2v (intermediate-affinity IL-2 variant fused to a mAb against fibroblast-activating protein [8]), PD1-IL2v (intermediate-affinity IL-2 variant fused to an anti-PD-1 mAb; bivalent) [8,9,11]), and/or checkpoint inhibition therapy (anti-PD-1 or anti-PD-L1 antibody)], or (B) upon regulatory T cell (Treg) depletion [3]. Selected genes represent the signature genes of better effector cells [8,11]. (C) Bar plots, constructed for illustration purposes only, showing the enrichment of KLRK1^+^ IL-7Rα^+^ (KILR) T cell signature genes (genes induced in activated CD8^+^ T cells upon Treg depletion) in splenic CD8^+^ T cells upon treatment with IL-2-based compounds and/or checkpoint inhibitors [3]. (A–C) Single cell RNA sequencing data were obtained from the following studies: Study 1 [8] (E-MTAB-11773), Study 2 [9] (GSE197854), Study 3 [11] (GSE206739), Study 4 [7], and Study 5 [3] (GSE183940). Abbreviations: NK, natural killer; LMCV, lymphocytic choriomeningitis virus; OVA, ovalbumin.

The gene expression profile induced in murine intratumoral and splenic CD8^+^ T cells upon IL-2 treatment in the above-mentioned models of cancer and chronic infection [7–9,11] is characterized by increased expression of genes encoding cytotoxic molecules (e.g., granzymes or cathepsins), adhesion molecules, receptors for proinflammatory cytokines and chemokines (IL-18R, IFNGR, and CCR5), transcription factors (e.g., TBET/*Tbx21*), interferon-response genes, NK receptors (killer cell lectin like receptor K1; KLRK1/NKG2D), and proinflammatory S100 proteins [22] (Figure 3A, data shown for illustrative purposes only). Collectively, these genes are associated with a strong cytotoxic response, suggesting that induction of this gene expression profile provides superior antitumor and antiviral killing properties to these better effector cells. Although it remains to be rigorously demonstrated, we propose that the formation of better effectors is a key mechanism mediating the potent antitumor effect of IL-2-based cancer immunotherapies in preclinical mouse models.

Although a better effector signature has been reported by three studies [8,9,11], in another study, PD1-IL2v treatment did not induce the better effector signature (NK receptors and cytotoxic molecules) in A20 lymphomas in BALB/C mice [7]. Although the differences among gene expression signatures upon various IL-2 treatments need to be explained by further studies, we suggest that the difference is caused by the monovalency of PD1-IL2v used in the latter study [7] (Figure 1C,D), indicating a low-avidity interaction of this molecule with target T cells. Moreover, the actual topology of the relative orientation of the IL-2v and anti-PD-1 antibody in the chimeric molecule might be important for the spatial assembly of IL-2v with the IL-2Rβγ on the cell surface, which also remains to be further tested.

The transcriptional profiles of CD8^+^ T cells from chronic LCMV infection in mice treated with WT IL-2 or with WT IL-2 plus anti-PD-1 antibody are largely similar, which suggests that the differentiation of CD8^+^ T cells into better effectors is induced by the IL-2 signal rather than by PD-1 blockade [11] (Figure 2C). Accordingly, treatment of C57BL/6 mice bearing B16F10 tumors with IL-2ICx selective for trimeric IL-2Rαβγ increased the frequency of GZMB^+^ and KLRK1/NGK2D^+^ cells (corresponding to the better effector T cells) among splenic and tumor-infiltrating CD8^+^ T cells evaluated by flow cytometry [3].

## Better effector T cells are clonally expanded antigen-specific cells

Only some CD8^+^ T cells differentiate into better effectors upon PD1-IL2v treatment (Figure 2A–C). Indeed, the preferential expansion of T cells specific to viral or tumor antigens, revealed as increased frequencies of CD8^+^ T cells binding the LCMV-specific or tumor-specific MHCI-tetramers in mouse models of chronic infections and cancer [7–9,11], has indicated that only antigen-stimulated CD8^+^ T cells form better effectors. Accordingly, better effectors are highly enriched in clonally expanded CD8^+^ T cells, as shown by the presence of multiple T cells with the same TCR in the better effector subset isolated from pancreatic tumors of PD1-IL2v-treated mice [8,9]. In some mouse tumor models, the therapeutic effects of IL-2-based treatment only manifest in combination with immunogenic chemotherapy (B16F10 melanoma in C57BL/6 mice and BCL1 leukemia in BALB/C mice) [3] or irradiation (K5L1940 adenocarcinoma in C57BL/6 mice) [10]. Most likely, the additional treatment might trigger the release and subsequent presentation of cancer antigens, which is required for better effector formation from tumor-specific T cells, although this remains conjectural.

## Strong IL-2 signals are required for the formation of better effectors

While treatment with WT IL-2 or PD1-IL2v increased the frequencies of better effectors in chronic LCMV infection or in Panc02-Fluc pancreatic adenocarcinoma, this effect was not observed upon treatment with IL-2v, which was not targeted to T cells [8,11] (Figure 2B). Hence, we propose that strong binding of IL-2 to its receptor, which is mediated via intact IL-2:IL-2Rα interaction or via anchoring of IL-2v to PD-1 in *cis*, is required to make better effector T cells. Accordingly, ‘synthetic effector’ T cells [i.e., genetically engineered chicken ovalbumin (OVA)-specific OT-I CD8^+^ T cells secreting nontargeted IL-2v and IL-33 alarmin] have potent antitumor activity in mice with implanted OVA-expressing B10 melanoma, but do not form better effectors [23]. This suggests that ‘synthetic effector’ T cells work via a different mechanism compared with PD1-IL2v treatment, perhaps based on the combination of stimulation of IL-2Rβγ by IL-2v, and activation of tumor-associated dendritic cells by IL-33:IL-33R interactions, although this remains to be further tested.

The better effector signature genes [8] appear to overlap with the profile of human and murine bystander activated CD8^+^ T cells stimulated by IL-15 (reviewed in [24]), a cytokine that binds to IL-2Rβγ and uses the identical signaling pathway to IL-2. IL-15-based biologicals represent another promising direction of experimental anticancer therapy (reviewed in [25]). Recently, modified IL-15 conjugated to an anti-PD-1 monoclonal antibody (PD1-IL15m) was shown to inhibit the growth of B16F10 or MC38 tumors in C57BL/6 mice in a dose-dependent manner by inducing proliferation (expression of *Mki67*) and cytotoxic effector gene expression (*Gzmb*, *Tbx21*, and *Ifng*) in CD8^+^ T cells [26]; the results showed a striking analogy between IL-2 and IL-15 targeted to PD-1^+^ T cells. Overall, IL-2 and IL-15 antitumor therapies might have a similar mode of action, which includes the formation of better effectors, although this remains to be further tested.

## T cell priming in the absence of Tregs can induce a similar gene expression program to IL-2-based therapy

It is well established that Tregs suppress effector T cell responses using multiple mechanisms (reviewed in [27]), mostly based on experiments in which Tregs suppressed activated CD4^+^ T cells. However, we argue that Tregs use IL-2 depletion as a dominant mechanism for the suppression of CD8^+^ T cells, as shown in several studies in mice [3,4,28,29]. These studies provide multiple layers of evidence for such a conclusion. First, IL-2 serum concentrations are increased in the absence of Tregs in mice [29]. Second, depletion of Tregs upregulates IL-2 signaling in activated CD8^+^ T cells in mice [3]. Third, although a high dose of IL-2 therapy causes Treg expansion in mice, it also induces effector CD8^+^ T cell differentiation and renders mice susceptible to CD8^+^ T cell-mediated experimental autoimmune diabetes to a similar extent to Treg depletion [3]. This would not be expected if Tregs used predominantly IL-2-independent mechanism(s) for CD8^+^ T cell suppression. Fourth, *Foxp3*^Cre^*Il2ra*^fl/fl^*Rosa26*^Stat5bCA^ mice with IL-2Rα-deficient Tregs (rescued by constitutive intracellular IL-2R signaling) develop hyperproliferation of CD8^+^, but not CD4^+^ T cells in lymph nodes [4]. However, additional mechanisms of Treg-mediated suppression of CD8^+^ T cells might also be important in particular contexts, warranting further investigation.

**Figure 4:**
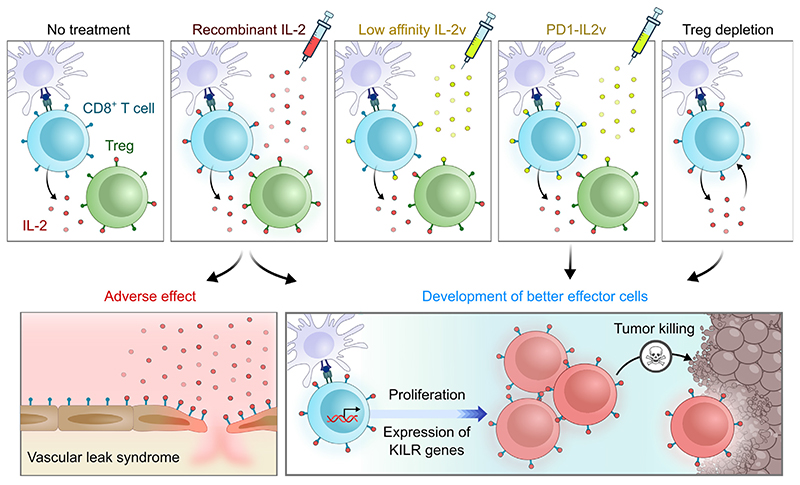
Key figure. Proposed mechanism of interleukin (IL)-2-based tumor clearance mediated by CD8^+^ T cells In this model, we propose that, under physiological conditions, antigen-activated CD8^+^ T cells produce the cytokine IL-2, which is largely sequestered by regulatory T cells (Tregs), expressing the high-affinity IL-2 receptor (IL-2R). High amounts of exogenous IL-2 may not only activate both Tregs and CD8^+^ T cells, but also lead to life-threatening adverse effects caused by damage to endothelial cells with subsequent vascular leak syndrome. Intermediate-affinity IL-2 variants (IL-2v) may bind preferentially to an intermediate-affinity IL-2R expressed by CD8^+^ T cells, but are not potent enough to trigger full activation *per se*. Novel molecules, represented by IL-2v fused to an antibody against inhibitory receptor PD-1 (PD1-IL2v), may act specifically on PD-1^+^ CD8^+^ T cells (enriched in tumor-specific cells), which proliferate and differentiate into better effector cells with superior anticancer cytotoxic activity. A similar effect might be achieved by Treg depletion, increasing IL-2 availability for CD8^+^ T cells. Abbreviation: KILR, KLRK1^+^IL-7Rα^+^CD8^+^ T cells.

In the absence of Tregs, OVA-specific OT-I CD8^+^ T cells form unusual effector KLRK1^+^IL-7Rα^+^ (KILR) CD8^+^ T cells after activation with their cognate antigen (intravenous injection of bone marrow-derived dendritic cells pulsed with OVA peptide) in C57BL/6 mice, as revealed by scRNAseq and flow cytometry [3]. Of note, KILR T cells resemble better effectors, as documented by their upregulation of better effector signature genes (Figure 3B) and, reciprocally, by the upregulation of KILR signature genes, such as *Klrk1*, *Ifitm1-3*, *Cd7*, and *Nkg7* in CD8^+^ T cells upon IL-2-based treatment (Figure 3C) [3,7–9,11]. Moreover, KILR T cells showed superior cytotoxic activity against adoptively co-transferred splenocytes loaded with cognate antigen in C57BL/6 mice [3]. Putative strong cytotoxicity was also proposed as a feature of better effectors [8]. Based on gene expression similarity and IL-2 dependency between KILR T cells and better effectors, we propose that these subsets may be related (Figure 4, Key figure) or even represent an identical subset, although this remains speculative.

## Stem-like cells are putative precursors of KILR and better effector T cells

KILR and better effector CD8^+^ T cells express IL-7Rα, the receptor for the prosurvival cytokine IL-7 [3,8,11]. This is paradoxical since: (i) IL-2 treatment *ex vivo* [30] or without antigenic activation *in vivo* [3] decreases the expression of IL-7Rα; and (ii) the expression of IL-7Rα is typical for memory, but not for effector T cells [31]. A possible explanation of IL-7Rα expression in KILR T cells is their putative origin from memory precursors rather than from effector T cells. Accordingly, the formation of KILR T cells by the above-described OT-I T cell priming in the absence of Tregs was accompanied by a decreased frequency of conventional TCF7^+^ stem-like memory precursor T cells, but not classical effector T cells, suggesting that TCF7^+^ stem-like precursor T cells are precursors of KILR T cells [3].

One study described intratumoral CD8^+^ T cells as the major target of PD1-IL2v therapy, because the inhibition of the T cell egress from the lymphoid tissues by FTY720 (fingolimod) did not impact the treatment efficacy of PD1-IL2v in a renal adenocarcinoma mouse model [7]. Similarly, a negligible effect of FTY720 administration was observed in a mouse model of B16F10 melanoma, when treated with PD1-IL15m [26]. Two studies proposed that better effectors were derived from CD8^+^ PD-1^+^ TCF7^+^ stem-like T cells, based on observations that PD1-IL2v expands CD8^+^ PD-1^+^ TCF7^+^ T cells in Panc02-H7-Fluc adenocarcinoma [8], in spontaneous pancreatic tumors of RIP1-Tag5 mice [9], and in mouse GL261 gliomas [9], validating this T cell subset as the putative target of this therapy. Overall, current evidence suggests that PD1-IL2v therapy induces the differentiation of better effectors from intratumoral TCF7^+^ stem-like T cells, which parallels the putative formation of KILR T cells from stem-like memory precursors in the spleen.

## The ‘exaggerated’ role of Tregs in IL-2-based cancer immunotherapy

The development of IL-2-based therapeutics has been accompanied by significant concern regarding its dual impact on tumor-specific T cells and immunosuppressive Tregs. Thus, IL-2v modifications have been developed to avoid/lower binding to the IL-2Rα subunit constitutively expressed on Tregs. However, clinical trials with these variants have not been successful, yet [16], perhaps because of their weak binding to the high-affinity IL-2Rαβγ expressed on activated CD8^+^ T cells.

Based on the above-mentioned model that Tregs might suppress CD8^+^ T cells via sequestering IL-2, we hypothesize that the concurrent stimulation of Tregs by IL-2-based biologicals does not pose a significant complication, since Tregs would not be able to suppress CD8^+^ T cells in the excess of exogenous IL-2R agonists. This is supported by experiments with mouse tumor models indicating that the administration of IL-2Rα-biased IL2ICx [32] or IL-2-IL-2Rα fusion protein preferentially stimulating IL-2Rα^+^ cells [33] can induce the potent antitumor activity of CD8^+^ T cells (B16F10 melanoma in C57BL/6 mice; and BCL1 leukemia and CT26 colon carcinoma in BALB/C mice) [3,34]. Moreover, antibody-mediated depletion of Tregs by anti-IL-2Rα antibody did not improve the survival of C57BL/6 mice bearing PK5L1940 adenocarcinoma that were treated with irradiation and PD1-IL2v [10], suggesting that Tregs did not efficiently suppress antitumor CD8^+^ T cells in response to the excess of exogenous IL-2R agonist. These observations are paradigm changing, since they challenge the scenario that IL-2-based biologicals targeting IL-2Rα^+^ T cells are immunosuppressive by stimulating Treg cells [35–41].

A large proportion of Tregs express PD-1, especially in tumors, such as human gastric cancer and nonsmall cell lung cancer [42,43]. Thus, PD1-IL2v might induce proliferation and boost a suppressive phenotype in intratumoral Tregs, although this has not been demonstrated. However, CD8^+^ T cells outnumbered Tregs in the tumor of PD1-IL2v-treated mice, as shown in the above-mentioned studies using pancreatic cancer and lymphoma models [7–10]; the resulting anti-cancer effect was evidenced by the prolonged survival and/or reduced tumor burden of mice. We propose a possible explanation for this phenomenon, whereby the intrinsic effect of PD1-IL2v on CD8^+^ T cells would be higher than that on Tregs, perhaps because of lower expression of PD-1 and/or or IL-2Rβ on Tregs, although this remains conjectural. A second hypothetical reason might be the resistance of CD8^+^ T cells to Treg-mediated inhibition upon IL-2 based therapy. In this scenario, Tregs might still use other mechanisms of suppression to regulate other cell types, such as effector CD4^+^ T cells [4], which can also contribute to tumor clearance [44,45].

Moreover, the effect of PD-1 blockade on Tregs upon PD1-IL2v treatment is unclear, since there is controversy over whether PD-1 signaling is a positive [46] or negative [43,47] regulator of Treg-mediated suppression, which further complicates the elucidation of the potential role of Tregs during PD1-IL2v therapy.

Overall, we argue that the significance of off-target stimulation of Tregs upon IL-2-based immunotherapy is likely not as serious an issue as conventionally believed, which would open new avenues for the development of novel candidate IL-2-based antitumor treatments. Certainly, this warrants robust investigation.

## Concluding remarks

Although the original idea of using IL-2 for therapeutic purposes is not new, we are currently experiencing a boom of different strategies using IL-2 for antitumor and antiviral therapies. In particular, these include the recent utilization of chimeric molecules of IL-2 and anti-PD-1 antibody, showing excellent efficacy in preclinical mouse models of cancer and chronic viral infection [7–11]. Single cell transcriptomics has revealed that strong IL-2R agonists can not only promote the differentiation of effector T cells, but also induce a unique gene expression profile in CD8^+^ T cells, which aligns with superior cytotoxic properties [8,9,11]. However, one of the biggest concerns in the design of IL-2-derived biologicals is avoidance of the concomitant stimulation of Tregs. This has motivated the design of IL-2 variants that do not act on Tregs [48–51], but suffer from low efficacy on CD8^+^ T cells [8,11]. Based on recent data, we propose that Tregs might not be able to suppress CD8^+^ T cell responses in the presence of strong exogenous IL-2R agonists and, thus, might not substantially mitigate the effects of IL-2-based therapy. However, their role might depend on particular immunological context, especially based on disease and tumor type, which is not fully understood currently. Another potential limitation of our proposed model is that the most of the underlying evidence has been generated in preclinical mouse models and it is not clear to what extent they apply to humans. Therefore, these and other open questions (see Outstanding questions) need to be resolved to bring optimal IL-2-derived treatments into the clinic, representing a fruitful area of future investigation.

## Highlights

The therapeutic potential of interleukin (IL)-2 in cancer and chronic infections has triggered the development of novel IL-2-based biologicals and combinatorial treatment strategies to achieve high efficacy and low off-target adverse effects in mouse models, before being tested in clinical trials.

Suboptimal IL-2 variants fused to an anti-PD-1 antibody specifically promote robust therapeutic effects in various preclinical cancer and infection mouse models (e.g., implanted lymphoma, pancreatic adenocarcinoma, glioma, colon carcinoma, spontaneous pancreatic cancer, and chronic viral infection). The effect occurs by targeting IL-2 to PD-1^+^ T cells and enhancing IL-2 binding to an ‘intermediate-affinity IL-2 receptor’ in *cis*.

Strong IL-2 signals are required to induce the formation of a unique differentiation state of antigen-stimulated CD8^+^ T cells, termed ‘better effectors’, with superior antitumor and antiviral properties in mice.

Based on preclinical mouse models, we propose that regulatory T cells suppress CD8^+^ T cells and the formation of ‘better effectors’ via IL-2 sequestration, which is disabled upon administration of exogenous IL-2-based biologicals.

## Significance

Strong IL-2 receptor stimulation of antigen-activated CD8^+^ T cells induces a unique gene expression program in which the cells harbor a superior cytotoxicity and anticancer/antiviral potential. This effect can be achieved by exogenous IL-2-based therapy or depletion of Tregs. Recent progress in the design of targeted IL-2-derived biologicals and single cell transcriptomics provide a novel framework for the development of putative therapies for treating cancer and chronic infections in humans.

## Box 1 The history of IL-2 therapies

The therapeutic potential of IL-2 was revealed ~40 years ago, when it was observed that *in vitro* IL-2-stimulated CD8^+^ T cells kill tumor cells [52,53]. A recombinant IL-2 therapy (aldesleukin) demonstrated clinical efficacy in metastatic renal cell carcinoma and melanoma, leading to its US Food and Drug Administration approval during the 1990s [54–56]. However, this therapy also caused severe adverse effects, including vascular leak syndrome, resulting from an off-target effect on endothelial cells [12,13,57].

The second generation of IL-2-based antitumor therapies featured drugs with modified binding to IL-2R aiming to stimulate cytotoxic CD8^+^ T cells and NK cells, but not endothelial cells or Tregs in mouse models and, subsequently, in clinical trials (NCT05267626^i^, NCT02983045^ii^, and NCT04855929^iii^) [18–20,48–51,58–69]. The respective strategies included modifications of the IL-2Rα binding site [48–51,58,59], selecting IL-2 variants with increased affinity to IL-2Rβ [18,60], complexes and fusion of IL-2 with anti-IL-2-antibodies [61–64] or IL-2Rα [65,66], or by designing novel IL-2-like drugs [67,68]. These innovative strategies resolved the adverse effects, but have not yet shown promising results in clinical trials [16], potentially due to their limited antitumor efficacy.

The third generation of IL-2-based antitumor drugs combined immune checkpoint blockade and specific IL-2 delivery to CD8^+^ PD-1^+^ T cells, which are found particularly in tumors, using a chimeric molecule of anti-PD-1 antibody fused to IL-2 variants binding specifically to intermediate-affinity receptor (IL-2Rβγ) (henceforth termed ‘intermediate-affinity IL-2 variants’). The promising effects of this approach in preclinical models of cancer and chronic infection [7–9] led to a current Phase 1 clinical trial (NCT04303858^iv^) (reviewed in [16]).

In contrast to antitumor therapy, low-dose IL-2 therapy can promote the expansion and differentiation of Tregs in humans [70,71] and, therefore, has been tested for safety and effectivity in Phase 2 clinical trials in type 1 diabetes mellitus (NCT01862120^v^) [72] and other autoimmune diseases (NCT01988506^vi^) [73].

Besides the rapid development of IL-2-based drugs for *in vivo* administration, IL-2 is routinely used ex *vivo* to stimulate cells for adoptive therapies, such as chimeric antigen receptor (CAR) T cells [74]. Moreover, T cells for adoptive transfer can be engineered *in vitro* to enhance their stimulation by IL-2 *in vivo*. One example is based on an engineered IL-2:IL-2Rβ pair, which enables using modified IL-2 to stimulate adoptively transferred T cells expressing the engineered IL-2Rβ, but not endogenous T cells [75]. A corresponding treatment using CAR-T cells reduced tumor burden, prolonged mouse survival, and exhibited lower toxicity compared with the systemic administration of WT IL-2 in two xenograft tumor models using immunodeficient NOD scid gamma (NSG) mice implanted with human Nalm6 leukemia cells [76] or human Raji lymphoma cells [77].

Overall, multiple IL-2-based approaches have been developed aiming to treat cancer, chronic infection, or autoimmunity, and over 100 clinical trials have been registered worldwide, suggesting that IL-2 is among the most promising candidates for immunotherapy [16].

## Glossary

### Abbreviations

Better effector: differentiation state of CD8^+^ T cells with superior antitumor and antiviral cytotoxic capacity; these develop *in vivo* after PD-1 blockade and IL-2 treatment.
Bystander-activated CD8^+^ T cells: CD8^+^ T cells, usually with memory phenotype, that become activated in an antigen-independent manner due to inflammatory signals during an immune response.
Chimeric antigen receptor (CAR) T cells: T cells engineered to express a synthetic receptor called a CAR. The CAR combines the antigen-binding domain of an antibody with TCR signaling components, enabling T cells to recognize specific antigens on target cells and initiate signaling and ensuing immune responses. CAR-T cells are used to treat various malignancies, mostly of hematopoietic origin.
*Cis* (interaction): in the context of receptor–ligand interactions, *cis* binding occurs when two molecules on the same cell interact with the ligand. By contrast, *trans* binding occurs when two molecules on different cells interact with the ligand.
FTY720 (fingolimod): sphingosine-1-phosphate receptor modulator, which induces the internalization of the receptor and, thus, prevents T cells from egressing the lymph nodes.
IL-2-based immunotherapies: therapeutic interventions that utilize IL-2 and its engineered variants to modify immune response to diseases, such as cancer and autoimmunity.
IL-2 immunocomplexes (IL2ICx): complexes of IL-2 and anti-IL-2 antibodies. The immunocomplexes have a longer *in vivo* half-life than IL-2 alone and thus, are potent immunomodulators of the IL-2 response. Based on the clone of the anti-IL-2 antibody, different IL-2 immunocomplexes have different in vivo effects (i.e., if the antibody blocks the IL-2Rα binding site, IL-2Rα-expressing cells are not preferentially stimulated).
IL-2 immunocytokines: engineered therapeutic molecules that combine IL-2 with specific monoclonal antibodies or antibody fragments to target immune cells expressing a particular cell surface receptor.
IL-2 mutein: mutated variant of the natural IL-2 cytokine. Through alterations in its amino acid sequence, it is designed to have modified functional properties, such as enhanced affinity for specific IL-2 receptors or reduced interactions with regulatory immune cells, while retaining the core immune-stimulating functions of IL-2.
IL-2 receptor (IL-2R): cell-surface protein complex that binds IL-2 and triggers the intracellular IL-2 signaling pathways. IL-2R has three subunits: IL-2Rα (alias CD25, intermediate-affinity IL-2 receptor, K_D_ ~10 nM), IL-2Rβ (alias CD122 or IL-15Rβ), and IL-2Rγ (alias CD132, common γ-chain). Naïve and memory T cells and NK cells express a dimeric IL-2Rβγ receptor with intermediate affinity to IL-2 (K_D_ ~1 nM). Tregs and recently activated T cells express a high-affinity trimeric IL-2Rαβγ receptor (K_D_ ~10 pM). One mechanism of Treg-mediated suppression of conventional T cells is using high-affinity receptors for sequestering IL-2 from the environment. IL-2R signaling induces the expression of IL-2Rα, which provides a positive feed-back loop.
IL-2 variant (IL-2v): modified version of IL-2 that has been engineered to alter its properties, especially the receptor binding. Here, we use IL-2v as a synonym for mutant IL-2, the binding of which to IL-2Rα is reduced or eliminated.
Immune checkpoint inhibitors: antibodies or other molecules that block inhibitory receptors, such as PD-1 or CTLA-4, on T cells, enhancing their proliferative capacity and/or effector function, mostly in the context of tumors. These inhibitors are widely used in cancer immunotherapy.
KILR T cell: differentiation state of CD8^+^ T cells that develops *in vivo* after antigenic stimulation in combination with supraphysiological IL-2R signals, occurring upon depletion of Tregs or administration of exogenous IL-2R agonists. It is characterized by expression of KLRK1, IL-7R, and other specific markers. KILR effectors and better effectors share striking gene expression similarities.
Killer cell lectin like receptor K1 (KLRK1/NKG2D): activating cell surface receptor expressed by NK cells and some CD8^+^ T cells. Its expression is characteristic of KILR^+^ effector and better effector cells.
Low-dose IL-2 therapy: administration of low doses of IL-2 in multiple dosing schedules aiming to suppress autoimmune responses in conditions such as type 1 diabetes mellitus, systemic lupus erythematosus, rheumatoid arthritis, and graft-versus-host disease, by expanding Treg cells.
NOD scid gamma (NSG) mice: mouse strain with a set of genetic mutations *(NOD.Cg-Prkdc^scid^ Il2rg^tm1Wjl^*/SzJ); develop severe combined immunodeficiency presenting with one of the most severe phenotypes without any mature T, B, or NK cells. Notably, NSG mice carry a complete null mutation in the gene encoding IL-2Rγ.
OT-I: transgenic OT-I mice produce monoclonal CD8^+^ T cells expressing OT-I TCRs specific to mouse H-2K^b^ MHC-I loaded with a chicken ovalbumin peptide (amino acid sequence is SIINFEKL). OT-I mice are commonly used for monitoring antigen-specific responses in mouse studies.
PD1-IL15m: fusion protein comprising an engineered variant of IL-15 that does not interact with IL-15Rα and that has a reduced affinity to IL2Rβγ, fused to the anti-PD-1 antibody.
PD1-IL2v: chimeric protein comprising an engineered variant of IL-2 not interacting with IL-2Rα fused to the anti-PD-1 antibody. It has superior antitumor effects compared with the IL-2v itself due to the increased *in vivo* half-life and *cis*-interaction with target T cells.
Single cell RNA sequencing (scRNAseq): technology used to quantify mRNA transcripts on a per cell basis, providing a high-resolution view of cell-to-cell variation in a biological sample.
Stem-like T cells: have the properties of stem cells (i.e., self-renewal potential and ability to differentiate into terminal effector T-cell stages: TCF7^+^). Stem-like properties are generally associated with memory T cells.
Synthetic effectors: genetically engineered CD8^+^ T cells with transgenic production of particular cytokines, which could be used in an adoptive transfer therapy to treat cancer. Synthetic effector CD8^+^ T cells producing IL-2v and IL-33 alarmin exhibited potent antitumor activity in a preclinical model.
TCF7: transcriptional factor that is a marker of T cell stemness; expressed in central memory T cells and stem-like T cells in chronic infection or in tumors. Also known as TCF-1.
Vascular leak syndrome: increase in vascular permeability causing the escape of blood plasma through capillary vessels into the tissue. This results in edema and subsequent tissue damage.

## Outstanding questions

How does strong IL-2 signaling induce a unique gene expression program leading to the formation of better effector CD8^+^ T cells? Although the proximal IL-2 signaling pathway is relatively well understood, it is unclear how strong IL-2 signals synergize with antigenic signaling to induce this transcriptional program leading to superior anti-cancer activity. Uncovering the respective signaling pathways leading to the triggering of the causative transcription factors and epigenetic regulators might reveal molecular mechanisms required for better effector formation that can be therapeutically induced or enhanced.

What is the role of Tregs in IL-2-based cancer immunotherapy? We posit that they might not be as crucial as once believed, especially in terms of their ability to suppress CD8^+^ T cells, but this should be thoroughly tested.

What is the optimal molecular structure of the PD1-IL2v chimera to provide strong targeted IL-2R signals and block PD-1:PD-L1 interactions? Different types of PD1-IL2v molecules appear to have different efficacies and potential to induce a better effector CD8^+^ T cell signature. Apparently, features such as anti-PD-1 antibody valency and topological orientation of fused proteins might have a role, but this has not been extensively investigated. Emerging PD1-IL2v drugs do not block PD-1 signaling completely and their efficacy can be improved in a combination with anti-PD-L1 antibodies. Thus, it might be possible to develop even more potent PD1-IL2 chimeric biologicals.

Is the effect of PD1-IL2v similar in humans? Since most of the emerging findings in IL-2-based immunotherapy are from preclinical mouse and humanized mouse models, the *in vivo* effects of IL-2-based therapy need to be addressed in humans, including the eventual formation of better effector human T cells.

## Resources

i
https://clinicaltrials.gov/study/NCT05267626

ii
https://clinicaltrials.gov/study/NCT02983045

iii
https://clinicaltrials.gov/study/NCT04855929

iv
https://clinicaltrials.gov/study/NCT04303858

v
https://clinicaltrials.gov/study/NCT01862120

vi
https://clinicaltrials.gov/study/NCT01988506

## References

[1] Malek TR, Castro I (2010). Interleukin-2 receptor signaling: at the interface between tolerance and immunity. Immunity.

[2] Au-Yeung BB (2017). IL-2 modulates the TCR signaling threshold for CD8 but not CD4 T cell proliferation on a single-cell level. J Immunol.

[3] Tsyklauri O (2023). Regulatory T cells suppress the formation of potent KLRK1 and IL-7R expressing effector CD8 T cells by limiting IL-2. Elife.

[4] Chinen T (2016). An essential role for the IL-2 receptor in T(reg) cell function. Nat Immunol.

[5] Hernandez R (2022). Engineering IL-2 for immunotherapy of autoimmunity and cancer. Nat Rev Immunol.

[6] Raeber ME (2022). Interleukin-2-based therapies in cancer. Sci Transl Med.

[7] Ren Z (2022). Selective delivery of low-affinity IL-2 to PD-1+ T cells rejuvenates antitumor immunity with reduced toxicity. J Clin Invest.

[8] Deak LC (2022). PD-1-cis IL-2R agonism yields better effectors from stem-like CD8(+) T cells. Nature.

[9] Tichet M (2023). Bispecific PD1-IL2v and anti-PD-L1 break tumor immunity resistance by enhancing stem-like tumor-reactive CD8(+) T cells and reprogramming macrophages. Immunity.

[10] Piper M (2023). Simultaneous targeting of PD-1 and IL-2Rbetagamma with radiation therapy inhibits pancreatic cancer growth and metastasis. Cancer Cell.

[11] Hashimoto M (2022). PD-1 combination therapy with IL-2 modifies CD8(+) T cell exhaustion program. Nature.

[12] Wylezinski LS, Hawiger J (2016). Interleukin 2 activates brain microvascular endothelial cells resulting in destabilization of adherens junctions. J Biol Chem.

[13] Krieg C (2010). Improved IL-2 immunotherapy by selective stimulation of IL-2 receptors on lymphocytes and endothelial cells. Proc Natl Acad Sci U S A.

[14] Nakagawa K (1996). Mechanisms of interleukin-2-induced hepatic toxicity. Cancer Res.

[15] Konrad MW (1990). Pharmacokinetics of recombinant interleukin 2 in humans. Cancer Res.

[16] Raeber ME (2023). A systematic review of interleukin-2-based immunotherapies in clinical trials for cancer and autoimmune diseases. Ebiomedicine.

[17] Waldman AD (2020). A guide to cancer immunotherapy: from T cell basic science to clinical practice. Nat Rev Immunol.

[18] Merchant R (2022). Fine-tuned long-acting interleukin-2 superkine potentiates durable immune responses in mice and non-human primate. J Immunother Cancer.

[19] Sharma M (2020). Bempegaldesleukin selectively depletes intratumoral Tregs and potentiates T cell-mediated cancer therapy. Nat Commun.

[20] Diab A (2021). Bempegaldesleukin plus nivolumab in first-line metastatic melanoma. J Clin Oncol.

[21] Spolski R (2018). Biology and regulation of IL-2: from molecular mechanisms to human therapy. Nat Rev Immunol.

[22] Xia C (2017). S100 Proteins as an important regulator of macrophage inflammation. Front Immunol.

[23] Corria-Osorio J (2023). Orthogonal cytokine engineering enables novel synthetic effector states escaping canonical exhaustion in tumor-rejecting CD8(+) T cells. Nat Immunol.

[24] Paprckova D (2023). Bystander activation in memory and antigen-inexperienced memory-like CD8 T cells. Curr Opin Immunol.

[25] Cai M (2023). Research progress of interleukin-15 in cancer immunotherapy. Front Pharmacol.

[26] Xu Y (2021). An engineered IL15 cytokine mutein fused to an anti-PD1 improves intratumoral T-cell function and antitumor immunity. Cancer Immunol Res.

[27] Schmidt A (2012). Molecular mechanisms of Treg-mediated T cell suppression. Front Immunol.

[28] Kastenmuller W (2011). Regulatory T cells selectively control CD8+ T cell effector pool size via IL-2 restriction. J Immunol.

[29] McNally A (2011). CD4+CD25+ regulatory T cells control CD8+ T-cell effector differentiation by modulating IL-2 homeostasis. Proc Natl Acad Sci U S A.

[30] Xue HH (2002). IL-2 negatively regulates IL-7 receptor alpha chain expression in activated T lymphocytes. Proc Natl Acad Sci U S A.

[31] Kaech SM (2003). Selective expression of the interleukin 7 receptor identifies effector CD8 T cells that give rise to long-lived memory cells. Nat Immunol.

[32] Spangler JB (2015). Antibodies to interleukin-2 elicit selective T cell subset potentiation through distinct conformational mechanisms. Immunity.

[33] Ward NC (2018). IL-2/CD25: a long-acting fusion protein that promotes immune tolerance by selectively targeting the IL-2 receptor on regulatory T cells. J Immunol.

[34] LaPorte KM (2023). Robust IL-2-dependent antitumor immunotherapy requires targeting the high-affinity IL-2R on tumor-specific CD8(+) T cells. J Immunother Cancer.

[35] Boyman O (2006). Selective stimulation of T cell subsets with antibody-cytokine immune complexes. Science.

[36] Tomala J, Kovar M (2016). IL-2/anti-IL-2 mAb immunocomplexes: a renascence of IL-2 in cancer immunotherapy?. Oncoimmunology.

[37] Webster KE (2009). In vivo expansion of T reg cells with IL-2-mAb complexes: induction of resistance to EAE and long-term acceptance of islet allografts without immunosuppression. J Exp Med.

[38] Spangler JB (2018). Engineering a single-agent cytokine/antibody fusion that selectively expands regulatory T cells for autoimmune disease therapy. J Immunol.

[39] Glassman CR (2021). Calibration of cell-intrinsic interleukin-2 response thresholds guides design of a regulatory T cell biased agonist. Elife.

[40] VanDyke D (2022). Engineered human cytokine/antibody fusion proteins expand regulatory T cells and confer autoimmune disease protection. Cell Rep.

[41] Trotta E (2018). A human anti-IL-2 antibody that potentiates regulatory T cells by a structure-based mechanism. Nat Med.

[42] Kumagai S (2022). Lactic acid promotes PD-1 expression in regulatory T cells in highly glycolytic tumor microenvironments. Cancer Cell.

[43] Kumagai S (2020). The PD-1 expression balance between effector and regulatory T cells predicts the clinical efficacy of PD-1 blockade therapies. Nat Immunol.

[44] Poncette L (2022). The role of CD4 T cells in rejection of solid tumors. Curr Opin Immunol.

[45] Brightman SE (2023). Neoantigen-specific stem cell memory-like CD4(+) T cells mediate CD8(+) T cell-dependent immunotherapy of MHC class II-negative solid tumors. Nat Immunol.

[46] Tan CL (2021). PD-1 restraint of regulatory T cell suppressive activity is critical for immune tolerance. J Exp Med.

[47] Kamada T (2019). PD-1(+) regulatory T cells amplified by PD-1 blockade promote hyperprogression of cancer. Proc Natl Acad Sci U S A.

[48] Carmenate T (2013). Human IL-2 mutein with higher antitumor efficacy than wild type IL-2. J Immunol.

[49] Charych DH (2016). NKTR-214, an engineered cytokine with biased IL2 receptor binding, increased tumor exposure, and marked efficacy in mouse tumor models. Clin Cancer Res.

[50] Chen X (2018). A novel human IL-2 mutein with minimal systemic toxicity exerts greater antitumor efficacy than wild-type IL-2. Cell Death Dis.

[51] Rosen DB (2022). TransCon IL-2 beta/gamma: a novel long-acting prodrug with sustained release of an IL-2Rbeta/gamma-selective IL-2 variant with improved pharmacokinetics and potent activation of cytotoxic immune cells for the treatment of cancer. J Immunother Cancer.

[52] Lotze MT (1981). Lysis of fresh and cultured autologous tumor by human lymphocytes cultured in T-cell growth factor. Cancer Res.

[53] Grimm EA (1982). Lymphokine-activated killer cell phenomenon. Lysis of natural killer-resistant fresh solid tumor cells by interleukin 2-activated autologous human peripheral blood lymphocytes. J Exp Med.

[54] Lotze MT (1985). In vivo administration of purified human interleukin 2. II. Half life, immunologic effects, and expansion of peripheral lymphoid cells in vivo with recombinant IL 2. J Immunol.

[55] Rosenberg SA (1987). A progress report on the treatment of 157 patients with advanced cancer using lymphokine-activated killer cells and interleukin-2 or high-dose interleukin-2 alone. N Engl J Med.

[56] Atkins MB (1999). High-dose recombinant interleukin 2 therapy for patients with metastatic melanoma: analysis of 270 patients treated between 1985 and 1993. J Clin Oncol.

[57] McKinstry KK (2019). Memory CD4 T cell-derived IL-2 synergizes with viral infection to exacerbate lung inflammation. PLoS Pathog.

[58] Gaggero S (2022). IL-2 is inactivated by the acidic pH environment of tumors enabling engineering of a pH-selective mutein. Sci Immunol.

[59] Ptacin JL (2021). An engineered IL-2 reprogrammed for anti-tumor therapy using a semi-synthetic organism. Nat Commun.

[60] Levin AM (2012). Exploiting a natural conformational switch to engineer an interleukin-2 ‘superkine’. Nature.

[61] Sahin D (2020). An IL-2-grafted antibody immunotherapy with potent efficacy against metastatic cancer. Nat Commun.

[62] Arenas-Ramirez N (2016). Improved cancer immunotherapy by a CD25-mimobody conferring selectivity to human interleukin-2. Sci Transl Med.

[63] Tomala J (2013). Chimera of IL-2 linked to light chain of anti-IL-2 mAb mimics IL-2/anti-IL-2 mAb complexes both structurally and functionally. ACS Chem Biol.

[64] Tomala J (2009). In vivo expansion of activated naive CD8+ T cells and NK cells driven by complexes of IL-2 and anti-IL-2 monoclonal antibody as novel approach of cancer immunotherapy. J Immunol.

[65] Lopes JE (2020). ALKS 4230: a novel engineered IL-2 fusion protein with an improved cellular selectivity profile for cancer immunotherapy. J Immunother Cancer.

[66] Lopes JE (2021). Pharmacokinetics and pharmacodynamic effects of nemvaleukin alfa, a selective agonist of the intermediate-affinity IL-2 receptor, in cynomolgus monkeys. J Pharmacol Exp Ther.

[67] Silva DA (2019). De novo design of potent and selective mimics of IL-2 and IL-15. Nature.

[68] Quijano-Rubio A (2023). A split, conditionally active mimetic of IL-2 reduces the toxicity of systemic cytokine therapy. Nat Biotechnol.

[69] Letourneau S (2010). IL-2/anti-IL-2 antibody complexes show strong biological activity by avoiding interaction with IL-2 receptor alpha subunit CD25. Proc Natl Acad Sci U S A.

[70] Seelig E (2018). The DILfrequency study is an adaptive trial to identify optimal IL-2 dosing in patients with type 1 diabetes. JCI Insight.

[71] Harris F (2023). IL-2-based approaches to Treg enhancement. Clin Exp Immunol.

[72] Rosenzwajg M (2020). Low-dose IL-2 in children with recently diagnosed type 1 diabetes: a Phase I/II randomised, double-blind, placebo-controlled, dose-finding study. Diabetologia.

[73] Rosenzwajg M (2019). Immunological and clinical effects of low-dose interleukin-2 across 11 autoimmune diseases in a single, open clinical trial. Ann Rheum Dis.

[74] Friedman KM (2018). Effective targeting of multiple B-cell maturation antigen-expressing hematological malignances by anti-B-cell maturation antigen chimeric antigen receptor T cells. Hum Gene Ther.

[75] Sockolosky JT (2018). Selective targeting of engineered T cells using orthogonal IL-2 cytokine-receptor complexes. Science.

[76] Zhang Q (2021). A human orthogonal IL-2 and IL–2Rbeta system enhances CAR T cell expansion and antitumor activity in a murine model of leukemia. Sci Transl Med.

[77] Aspuria PJ (2021). An orthogonal IL-2 and IL-2R beta system drives persistence and activation of CART cells and clearance of bulky lymphoma. Sci Transl Med.

